# A Bird’s Eye View on the Origin of Aortic Hemogenic Endothelial Cells

**DOI:** 10.3389/fcell.2020.605274

**Published:** 2020-11-17

**Authors:** Pedro Seco, Gabriel G. Martins, António Jacinto, Ana Teresa Tavares

**Affiliations:** ^1^iNOVA4Health, CEDOC, NOVA Medical School, Universidade Nova de Lisboa, Lisbon, Portugal; ^2^Instituto Gulbenkian de Ciência, Oeiras, Portugal; ^3^Faculdade de Ciências, Universidade de Lisboa, Lisbon, Portugal

**Keywords:** hemogenic endothelium, hemangioblast, dorsal aorta, yolk sac, avian embryo, lineage-tracing

## Abstract

During early embryogenesis, the hemogenic endothelium of the developing dorsal aorta is the main source of definitive hematopoietic stem cells (HSCs), which will generate all blood cell lineages of the adult organism. The hemogenic endothelial cells (HECs) of the dorsal aorta are known to arise from the splanchnic lateral plate mesoderm. However, the specific cell lineages and developmental paths that give rise to aortic HECs are still unclear. Over the past half a century, the scientific debate on the origin of aortic HECs and HSCs has largely focused on two potential and apparently alternative birthplaces, the extraembryonic yolk sac blood islands and the intraembryonic splanchnic mesoderm. However, as we argue, both yolk sac blood islands and aortic HECs may have a common hemangioblastic origin. Further insight into aortic HEC development is being gained from fate-mapping studies that address the identity of progenitor cell lineages, rather than their physical location within the developing embryo. In this perspective article, we discuss the current knowledge on the origin of aortic HECs with a particular focus on the evidence provided by studies in the avian embryo, a model that pioneered the field of developmental hematopoiesis.

## Introduction

**FIGURE 1:**
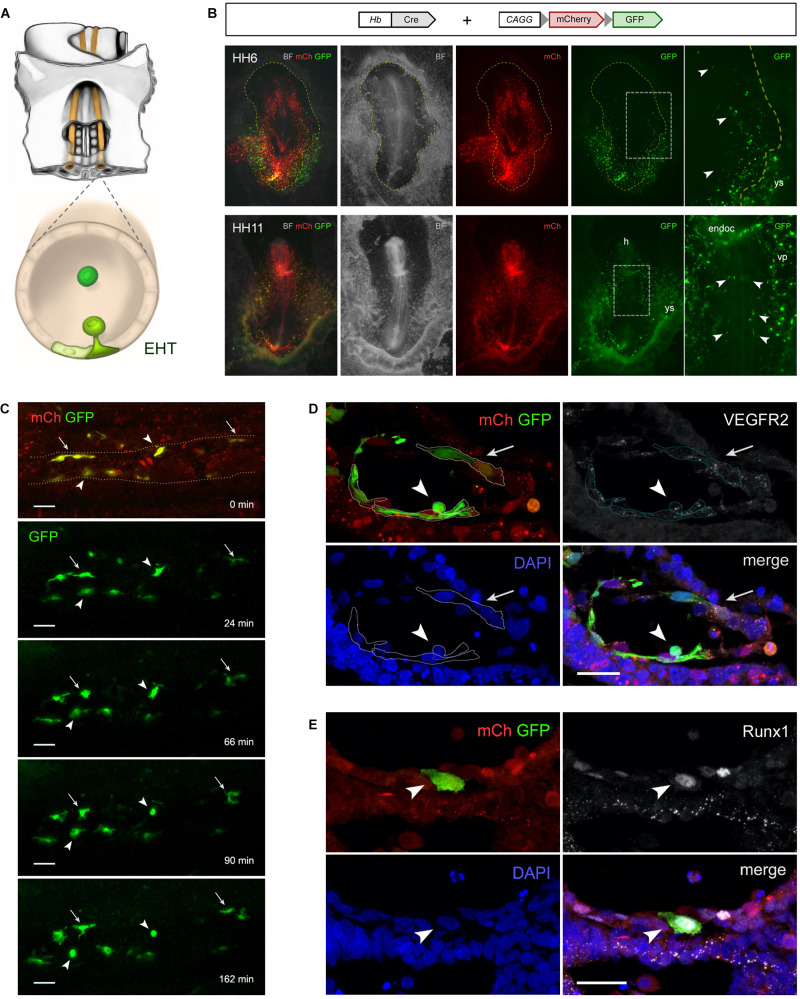
Hemangioblasts give rise to aortic hemogenic endothelial cells in the chick embryo. **(A)** Illustration of the central region of a stage HH11 chick embryo (ventral side up) highlighting the paired dorsal aortae (orange) and the hemogenic endothelial cells (HECs; green) localized in the aortic floor that differentiate into hematopoietic stem/progenitor cells via an endothelial-to-hematopoietic transition (EHT). **(B–E)** Hb-Cre and pCAGG-LoxP-mCherry-LoxP-GFP plasmids were used to analyze the progeny of chick hemangioblasts. Chick embryos were electroporated *ex ovo* at stage HH3 using an Intracel TSS20 electroporator, incubated until stage HH13 in New culture (New, 1955) or until stage HH16 in MC culture (Nagai et al., 2011), and imaged either as whole mounts using a Zeiss SteREO Lumar stereomicroscope **(B)** and a Prairie Multiphoton system **(C)**, or in immunolabeled cryosections using a Zeiss LSM710 confocal microscope **(D,E)**. **(B)** While mCherry (mCh) is ubiquitously expressed in all electroporated cells, GFP expression is specifically detected in hemangioblasts and their progeny. mCh expression in GFP+ cells may result from the presence of unrecombined copies of the reporter construct and/or persistent mCh transcripts and protein. At stage HH6 (*n* = 36; top), GFP+ hemangioblasts are found in both the yolk sac (ys) and the intraembryonic region (arrowhead; dashed yellow line outlines the embryo). At stage HH11 (*n* = 52; bottom), GFP+ hemangioblast-derived cells are found in the yolk sac (ys), head region (h), endocardium (endoc), intraembryonic vascular plexus (vp) and dorsal aorta (arrowheads). **(C)** Time-lapse images of electroporated HH11–12 chick embryo showing the dynamics of GFP+ hemangioblast-derived cells in the dorsal aorta endothelium (*n* = 3; see Video S1 and legend). During the imaging period (168 min), two GFP+ HECs undergo EHT (arrowheads), whereas other GFP+ cells remain as endothelial cells (arrows). **(D,E)** Transverse sections through the dorsal aortae of electroporated chick embryos immunolabeled with primary antibodies against GFP (Roche, 11814460001; Invitrogen, A11122; green), VEGFR2 (gift from Anne Eichmann; Eichmann et al., 1997; *n* = 5; **D**; HH13; white) or Runx1 (Abcam, ab92336; *n* = 6; **E**; HH16; white), and secondary antibodies with Alexa Fluor 488 (Invitrogen, A11001 and A11008) or Alexa Fluor 647 (Jackson ImmunoResearch, 715-605-151 and 711-605-152). Cell nuclei were labeled with DAPI (Sigma-Aldrich; blue). Electroporated cells are identified by mCh fluorescence (red). **(D)** GFP+ cells co-express VEGFR2, a marker of endothelial cells, and exhibit either a HEC morphology (arrowhead) or a typical endothelial cell shape (arrow). **(E)** Prior to emergence from the aortic floor, a GFP+ cell can be identified as a HEC by Runx1 expression (arrowhead), a marker of endothelial cells with hemogenic potential. BF, brightfield; *n*, number of embryos.

Hemogenic endothelial cells (HECs) are specialized vascular endothelial cells with the potential to give rise to hematopoietic stem/progenitor cells (HSPC) during vertebrate embryogenesis (Jaffredo et al., 1998; Zovein et al., 2008; Gritz and Hirschi, 2016). During this differentiation process, known as endothelial-to-hematopoietic transition (EHT), HECs gradually round up, separate from their neighboring cells and bud off from the endothelium (Eilken et al., 2009; Lancrin et al., 2009; Bertrand et al., 2010; Boisset et al., 2010; Kissa and Herbomel, 2010; Lam et al., 2010; Ottersbach, 2019; Figure 1A). Depending on developmental stage and location, HECs differentiate into HSPC populations with different hematopoietic potential. In mammalian and avian embryos, the first HECs are observed in the yolk sac blood islands and give rise to erythro-myeloid progenitors (EMPs) (Li et al., 2005; McGrath et al., 2015; Frame et al., 2016) and lymphoid progenitors (Yoshimoto et al., 2011, 2012). In addition, HECs located in the endocardium only generate EMPs (Nakano et al., 2013), whereas those in the head arteries and vitelline/umbilical arteries generate both EMPs and definitive hematopoietic stem cells (HSCs), the founders of adult hematopoietic cells (De Bruijn et al., 2000; Zovein et al., 2010; Li et al., 2012). Yet, HSC-producing HECs reside mainly in the ventral wall of the dorsal aorta in all vertebrate embryos studied to date (Medvinsky and Dzierzak, 1996; Jaffredo et al., 1998; Ciau-Uitz et al., 2000; Oberlin et al., 2002; Zovein et al., 2008; Bertrand et al., 2010). Recent studies suggest that aortic HECs retain a dual potential for differentiation into endothelial or hematopoietic cells (Hou et al., 2020), which may be driven toward a hemogenic fate by local cues that promote their detachment from the endothelium and cell cycle re-entry (Yue et al., 2012; Zhang et al., 2014; Canu et al., 2020).

In contrast to their clear developmental fate, the origin of aortic HECs and HSCs remains a matter of active debate. The enduring question is whether aortic HECs arise from extraembryonic yolk sac-derived progenitors that migrate into the developing aorta or from local intraembryonic progenitors, with experimental evidence supporting both possibilities (reviewed in Medvinsky et al., 2011; Wittamer and Bertrand, 2020). The seemingly contradictory results may be explained by the use of different models, time frames and methods. Namely, the evaluation of HSC differentiation potential in *in vitro* culture and transplantation assays was used to verify the presence of HSC precursor cells in different embryonic tissues. Data from these studies mostly support an intraembryonic origin for aortic HECs (Medvinsky and Dzierzak, 1996; Cumano et al., 2001; Yvernogeau and Robin, 2017). However, differentiation potentials may not reflect the original developmental fate of the explanted cell populations, which is largely influenced by their natural microenvironment and commitment status. Alternatively, the origin of aortic HECs has been more accurately investigated using fate-mapping and live imaging methods. In particular, lineage-tracing experiments using tamoxifen-inducible mouse models have indicated that yolk sac-derived cells migrate into the embryo and give rise to aortic HECs (Samokhvalov et al., 2007; Tanaka et al., 2014). However, conclusions from these studies may be compromised by the persistence of tamoxifen in the system and consequent labeling of intraembryonic cells (Senserrich et al., 2018). In addition to mouse models, fate-mapping studies in avian embryos have also greatly contributed to the discussion. For more than a century, observations in avian embryo models have led to major fundamental discoveries in the field of hematopoietic development (Le Douarin and Dieterlen-Lièvre, 2013; Jaffredo and Yvernogeau, 2014). After a brief description of yolk sac and dorsal aorta development, this perspective article will review and discuss the experimental evidence in chick and quail embryos that provided key insight into the origin of aortic HECs and vastly enriched our understanding of hematopoietic development.

## Yolk Sac and Dorsal Aorta Development in the Avian Embryo

To better interpret the evidence supporting each of the two proposed sites of origin of aortic HECs, the yolk sac blood islands (extraembryonic) and the prospective ventral dorsal aorta (intraembryonic), it is important to understand when and how these tissues develop. Both the yolk sac blood islands and the ventral endothelium of the dorsal aorta derive from the splanchnic lateral plate mesoderm, which, together with the endoderm, forms the splanchnopleure (Prummel et al., 2020). During gastrulation, these mesodermal cells arise from median-posterior sections of the primitive streak, with the posterior region giving rise to more lateral (extraembryonic) tissues (Psychoyos and Stern, 1996). In addition to their common mesodermal origin, the developmental paths of the yolk sac and dorsal aorta are closely coordinated in time and space, thus ensuring the proper establishment of embryonic blood circulation.

The yolk sac blood islands give rise to the extraembryonic vascular network and to the first hematopoietic cells of the developing embryo (Sabin, 1920). These structures are formed by a subpopulation of mesodermal-derived cells known as hemangioblasts, the precursors of both endothelial and hematopoietic cell lineages (Sabin, 1920; Murray, 1932). In the chick embryo, hemangioblasts arise in the yolk sac at Hamburger and Hamilton (HH) stage 5–6 (Hamburger and Hamilton, 1992), where they aggregate into blood islands at stage HH7–8 and start to differentiate into endothelial and hematopoietic cells at stage HH9–10 (Nagai et al., 2018). It is currently thought that these hematopoietic cells originate from two types of hemangioblast-derived progenitors: primitive hematopoietic cells derive from hemogenic angioblasts, whereas EMPs derive from hemogenic endothelial cells (Lacaud and Kouskoff, 2017). Proximal blood islands differentiate only into endothelial cells that will form a connecting network between the extraembryonic vasculature and the intraembryonic vascular plexus (Coffin and Poole, 1988; Nagai et al., 2018). At stages HH9 to HH11, this vascular plexus is contiguous with the developing endocardium (anterior region) and dorsal aorta (posterior region) and will later give rise the vitelline veins and arteries (le Noble et al., 2004).

The dorsal aorta is not only the first and largest intraembryonic blood vessel, but also an important site of secondary hematopoiesis (reviewed in Medvinsky et al., 2011). In avian embryos, paired dorsal aortae arise at stage HH8 as bilateral longitudinal cords of endothelial precursor cells (or angioblasts), which are derived from the splanchnic mesoderm (Poole and Coffin, 1989; Pardanaud et al., 1996). At stage HH9, angioblasts start to coalesce and remodel into two endothelial vessels. A few hours later (stage HH10), the two vessels move toward the ventral midline, where they will fuse into a single tube (stage HH13) (Pardanaud et al., 1987; Coffin and Poole, 1988). During these stages, somite-derived endothelial cells are integrated into the dorsal region (roof) of the dorsal aorta and gradually displace the splanchnic mesoderm-derived endothelial cells to the ventral region (floor), which is where HECs arise (Pardanaud et al., 1996; Jaffredo et al., 2013). In the caudal region of the embryo, the dorsal aorta elongates posteriorly and becomes attached to the intraembryonic vascular plexus, thus forming a connection with the extraembryonic vascular network (Pardanaud et al., 1987; Coffin and Poole, 1988). Circulating blood cells can be seen in the dorsal aorta by stage HH12 (early day 2 of development), shortly after heartbeat onset at stage HH10–11 (Hogers et al., 1995). From then on, blood circulation between the yolk sac and the embryo body is established, which makes it impossible to determine if aortic HECs that may arise hereafter have an extra- or intraembryonic origin.

## Tracing the Origin of Avian Aortic HECs

Since aortic HECs arise prior to the establishment of circulation, their progenitor cells are thought to either migrate into the presumptive ventral aorta region from the adjacent yolk sac (extraembryonic origin) or develop *in situ* within the presumptive dorsal aorta region (intraembryonic origin) (Medvinsky et al., 2011; Wittamer and Bertrand, 2020). Both hypotheses have gathered support over the years from fate-mapping studies using classical grafting techniques as well as more recent genetic and time-lapse imaging methods in electroporated or transgenic avian embryos.

Early experimental studies addressed the origin of definitive HSCs before the identification of aortic HECs as their progenitor cells (Cormier and Dieterlen-Lièvre, 1988; Jaffredo et al., 1998). The colonization of developing hematopoietic organs by yolk sac-derived HSCs was initially suggested by studies using parabiotic chick embryos (Moore and Owen, 1965). Yet, these experiments evaluated only the contribution of circulating cells (at 6–8 days of development), suggesting that embryo-derived HSCs might be already present in the yolk sac. The intraembryonic origin of HSCs was originally proposed in a series of classical grafting studies using avian yolk sac chimeras (reviewed in Jaffredo and Yvernogeau, 2014). The first experimental evidence came from analyzing chimeras of quail embryo bodies grafted onto chick yolk sacs before circulation is established. In most chimeras, the dorsal aorta (Dieterlen-Lièvre and Martin, 1981), spleen and thymus (Dieterlen-Lièvre, 1975) contained exclusively quail cells, thus indicating that the adult hematopoietic system was derived from the embryo proper and not from the yolk sac. The same conclusion was reached in studies of homospecific yolk sac chimeras from different-sex or allogeneic chick embryos (Lassila et al., 1978, 1982; Martin et al., 1978; Beaupain et al., 1979). Nonetheless, since the earliest stage of grafting was HH9 (Dieterlen-Lièvre, 1975; Lassila et al., 1978; Beaupain et al., 1979; Dieterlen-Lièvre and Martin, 1981), the possibility exists that yolk sac-derived angioblasts (or hemangioblasts) have colonized the presumptive dorsal aorta region before this stage.

The hypothesis that hemangioblasts give rise to aortic endothelial cells is supported by evidence showing that dorsal aorta-forming angioblasts express the hemangioblast and endothelial/blood marker Tal1/Scl (Drake et al., 1997) and that extraembryonic cells migrate to and contribute to the dorsal aorta endothelium (Sato et al., 2010; Tanaka et al., 2014; Eliades et al., 2016). However, as mentioned earlier, developmental cell fate is best addressed with *in vivo* lineage-tracing and time-lapse imaging methods, which first require the identification of a lineage-specific marker capable of labeling a particular cell population and its progeny (Stern and Fraser, 2001). Indeed, we have identified a hemangioblast enhancer (Hb) that is able to specifically activate the expression of a reporter gene (eGFP) in chick embryo hemangioblasts as they ingress through the posterior primitive streak at stages HH3 to HH6 (Teixeira et al., 2011). The Hb-eGFP reporter was used to study the dynamics of blood island morphogenesis in live imaging assays (Teixeira et al., 2011), and to isolate and characterize the gene expression profile of chick embryo hemangioblasts (Serrado Marques et al., 2018). Moreover, Zamir et al. (2017) used this reporter to show that a subpopulation of Hb-eGFP+ hemangioblasts gives rise to hemogenic angioblasts that can be found in both the extraembryonic yolk sac and intraembryonic lateral plate mesoderm at stage HH7, and that contribute to the hemogenic endothelium of the dorsal aorta. However, although eGFP RNA and protein stability may enable the detection of Hb-eGFP+ hemangioblast-derived cells, lineage-tracing analysis is required to accurately identify the progeny of hemangioblasts.

In order to label and trace the hemangioblast lineage, we took advantage of a Cre-Lox system in which a plasmid containing the hemangioblast enhancer driving Cre recombinase expression (Hb-Cre) and the conditional reporter plasmid pCAGG-LoxP-mCherry-LoxP-GFP are co-electroporated into chick embryos (stage HH3), as previously reported for neuronal lineages (Avraham et al., 2009). In this system, cells that express Cre under the control of the Hb enhancer will recombine out the floxed mCherry sequence and activate GFP expression, enabling the identification of hemangioblasts as well as their progeny. We observed that GFP+ cells are present in both the yolk sac and intraembryonic lateral plate mesoderm at early stages (Figure 1B, top), which supports the existence of a subpopulation of intraembryonic hemangioblasts. At later stages, GFP+ hemangioblast-derived cells are detected in the yolk sac blood islands, head region, endocardium, intraembryonic vascular plexus and dorsal aorta (Figure 1B, bottom). Using time-lapse multiphoton microscopy imaging of electroporated chick embryos, we observed that some GFP+ cells in the dorsal aorta undergo EHT at stages HH11–12 (Supplementary Video S1 and Figure 1C). GFP+ cells in the aortic endothelium display either a flat endothelial morphology or HEC features, as seen also in cross-sections of the dorsal aorta labeled for VEGFR2, a marker of endothelial cells (Eichmann et al., 1993; Figure 1D), and for Runx1, a marker of HECs (Jaffredo et al., 2005; Figure 1E). Of note, Runx1 expression is also detected in some mCherry+GFP-cells, suggesting that hemangioblasts are not the exclusive source of aortic hemogenic endothelium. Taken together, these observations indicate that hemangioblast-derived hemogenic angioblasts give rise to a subpopulation of aortic HECs in the chick embryo. However, further experiments are required to reveal the hematopoietic potential of hemangioblast-derived aortic HECs. Our findings are consistent with lineage-tracing experiments in the mouse embryo showing that extraembryonic Runx1+ hemogenic angioblasts migrate into the intraembryonic region prior to circulation and contribute to the dorsal aorta hemogenic endothelium (Tanaka et al., 2014). It is therefore, conceivable that both yolk sac blood islands and aortic HECs originate from hemangioblasts.

**FIGURE 2:**
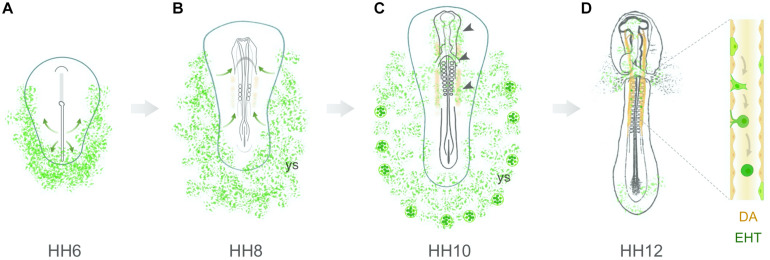
Hemangioblastic origin of aortic hemogenic endothelial cells in the early chick embryo. **(A)** Hemangioblasts arise from lateral plate mesodermal cells that ingress through the posterior region of the primitive streak. At stage HH6, these cells are located in the lateral and posterior regions of the embryo, both in the yolk sac and intraembryonic region. **(B)** At stage HH7–8, hemangioblasts aggregate to form blood islands within the yolk sac (ys). Concomitantly, a subpopulation of hemangioblast-derived cells (or angioblasts) starts to migrate toward the presumptive paired dorsal aortae (orange). **(C)** At stage HH10, yolk sac hemangioblasts have already started to differentiate into endothelial and hematopoietic cells in the distal blood islands. Inside the embryo, hemangioblast-derived cells are found in the head region, endocardium, vascular plexus and dorsal aortae (arrowheads). **(D)** At stages HH11–12, hemangioblast-derived HECs are detected in the dorsal aortae (DA), where they undergo an endothelial-to-hematopoietic transition (EHT).

In summary, recent evidence suggests that hemangioblasts colonize the presumptive dorsal aorta region (stages HH7–8), contribute to the developing aortic endothelium (stages HH9–10) and give rise to aortic HECs (stage HH11 onwards; Figure 2). Yet, it remains to be determined if these hemangioblasts are originally located in the intraembryonic splanchnic mesoderm (as shown in Figure 1B, top; Zamir et al., 2017) or if they migrate medially from the yolk sac at stages HH7–8. In any case, this early population of aortic HECs progenitors would be reasonably considered to have an intraembryonic origin in grafting studies of yolk sac chimeras established at stage HH9 and later. In conclusion, aortic HECs are likely to originate from multiple sources that combine hemangioblasts and other splanchnic mesoderm-derived hemogenic progenitors.

Further evidence supporting a hemangioblastic origin for aortic HECs was provided by studies in *Xenopus* embryos. Lineage tracing and co-expression analysis of endothelial and blood markers demonstrated that HSC-producing aortic HECs arise from hematovascular progenitors known as adult or definitive hemangioblasts (Walmsley et al., 2002; Ciau-Uitz et al., 2013). Definitive hemangioblasts arise from lateral plate mesodermal cells that express Tal1/Scl and lie close to the somites. These features are also typical of aortic HEC precursors in zebrafish, chick and mouse, suggesting that definitive hemangioblasts may indeed exist in all vertebrates (reviewed in Ciau-Uitz and Patient, 2016). Furthermore, converging evidence has come from *in vitro* differentiation studies of human embryonic stem cells and induced pluripotent stem cells, which show that definitive HSCs-producing HECs can be derived from hemangioblastic progenitors (reviewed in Chen et al., 2015). Ultimately, this information can be used to develop hemangioblast-based differentiation protocols for the *in vitro* generation of transplantable HSCs.

## Concluding Remarks

The hemogenic endothelium of the developing dorsal aorta is considered to be the major site of HSC production in the early embryo. As such, aortic HECs have been the subject of extensive research on many of their biological aspects, such as molecular signature, differentiation potential and developmental origin. For over 40 years, the extra- vs. intraembryonic origin of HSCs and aortic HECs has been the focus of numerous studies, for which the avian embryo was a pioneer model. However, classical grafting experiments in avian embryos need to be reinterpreted in light of recent lineage-tracing evidence demonstrating that hemangioblasts give rise to aortic HECs at early developmental stages. This finding is particularly relevant in the context of *in vitro* differentiation of pluripotent stem cells into HSCs for clinical applications.

## Data Availability Statement

The original contributions presented in the study are included in the article/Supplementary Material, further inquiries can be directed to the corresponding author.

## Ethics Statement

Ethical review and approval was not required for the animal study because all experimental procedures were performed in chicken embryos with less than 3 days of development, which are not considered experimental animal subjects according to the Portuguese Law (Decree-Law 113/2013) and European Guidelines (Directive 2010/63/EU).

## Author Contributions

PS performed the experiments and contributed to the manuscript writing. GM helped with the research design, image acquisition, and data analysis. AJ contributed to the data interpretation and scientific discussion. AT designed and performed the experiments, analyzed and interpreted the data, and wrote the manuscript. All authors contributed to manuscript revision and approved the final version.

## Conflict of Interest

The authors declare that the research was conducted in the absence of any commercial or financial relationships that could be construed as a potential conflict of interest.

## Supplementary Material

Supplementary Video 1 Hemangioblast-derived hemogenic endothelial cells are detected in the chick embryo dorsal aorta. Time-lapse movie of a stage HH11–12 live chick embryo in New culture electroporated at stage HH3 with Hb-Cre and pCAGG-LoxP-mCherry-LoxP-GFP reporter constructs showing a portion of the dorsal aorta (still frames in Figure 1C). The anterior side is to the left. Brightfield images were acquired on a Zeiss SteREO Lumar stereomicroscope (initial frames). Time-lapse 4D stacks were acquired on a Prairie Multiphoton system equipped with GaAsP detectors mounted on an Olympus BX61 microscope equipped with a 10× 0.3NA objective, and the datasets were processed and 3D reconstructed using Fiji (ImageJ) and Imaris software (Bitplane). Red represents mCherry, and green, GFP expression. Arrowheads point at two GFP+ hemogenic endothelial cells that round up and emerge from the aortic wall. Arrows point at GFP+ cells that maintain an endothelial morphology. Time is indicated in minutes in the lower right corner.
